# Reliability and Validity of Selected PROMIS Measures in People with Rheumatoid Arthritis

**DOI:** 10.1371/journal.pone.0138543

**Published:** 2015-09-17

**Authors:** Susan J. Bartlett, Ana-Maria Orbai, Trisha Duncan, Elaine DeLeon, Victoria Ruffing, Katherine Clegg-Smith, Clifton O. Bingham

**Affiliations:** 1 Department of Medicine, Divisions of Clinical Epidemiology, Rheumatology, and Respiratory Epidemiology, McGill University / McGill University Health Centers, Montreal, QC, Canada; 2 Department of Medicine, Division of Rheumatology, Johns Hopkins School of Medicine, Baltimore, MD, United States of America; 3 Bloomberg School of Public Health, Center for Qualitative Studies in Health and Medicine, Johns Hopkins University, Baltimore, MD, United States of America; University of Texas Southwestern Medical Center, UNITED STATES

## Abstract

**Purpose:**

To evaluate the reliability and validity of 11 PROMIS measures to assess symptoms and impacts identified as important by people with rheumatoid arthritis (RA).

**Methods:**

Consecutive patients (N = 177) in an observational study completed PROMIS computer adapted tests (CATs) and a short form (SF) assessing pain, fatigue, physical function, mood, sleep, and participation. We assessed test-test reliability and internal consistency using correlation and Cronbach’s alpha. We assessed convergent validity by examining Pearson correlations between PROMIS measures and existing measures of similar domains and known groups validity by comparing scores across disease activity levels using ANOVA.

**Results:**

Participants were mostly female (82%) and white (83%) with mean (SD) age of 56 (13) years; 24% had ≤ high school, 29% had RA ≤ 5 years with 13% ≤ 2 years, and 22% were disabled. PROMIS *Physical Function*, *Pain Interference* and *Fatigue* instruments correlated moderately to strongly (rho’s ≥ 0.68) with corresponding PROs. Test-retest reliability ranged from .725–.883, and Cronbach’s alpha from .906–.991. A dose-response relationship with disease activity was evident in *Physical Function* with similar trends in other scales except *Anger*.

**Conclusions:**

These data provide preliminary evidence of reliability and construct validity of PROMIS CATs to assess RA symptoms and impacts, and feasibility of use in clinical care. PROMIS instruments captured the experiences of RA patients across the broad continuum of RA symptoms and function, especially at low disease activity levels. Future research is needed to evaluate performance in relevant subgroups, assess responsiveness and identify clinically meaningful changes.

## Introduction

There is growing recognition of the importance of placing patients at the center of healthcare by developing patient-centered care models and integrating patient-valued outcomes into shared decision-making [1, 2]. Patient-reported outcomes (PROs) contribute essential information from the perspective of people living with a chronic disease and its treatments about the status of or a change in their physical, emotional, and social health outcomes [3].

Arthritis is the leading cause of disability in the US [4]. Rheumatoid arthritis (RA), the most common form of inflammatory arthritis, is a painful, disabling, and destructive disease that greatly impairs quality of life and shortens the lifespan [5–7]. RA cannot be cured and everyday life with RA is strongly influenced by symptoms that fluctuate widely and have far-reaching impacts on physical, mental, and social health [8, 9]. In RA, three PROs have been included within the American College of Rheumatology core set of outcome measures recommended for use in randomized clinical trials (RCTs) [10] and clinical care [11] including global ratings of disease activity or health, pain, and physical function; more recently, fatigue also has been recommended for inclusion [10, 12, 13]. However, other symptoms and impacts of importance to RA patients include stiffness, sleep disturbance, emotional distress, and participation in life activities [14–18]. Also, with the goal of RA treatment now remission or low disease activity (LDA), it is important to be able to monitor subtle improvements and worsening of symptoms and function [18–20].

The process for developing and validating PROs has evolved considerably over the last two decades and now includes recommendations to identify patient-relevant symptoms and impacts through rigorous qualitative and psychometric processes and demonstrate validity in the targeted patient population and context of intended use (e.g. RCT vs. clinical practice) [21–23]. As instruments from RCTs are adopted for use in”real world” settings and pragmatic clinical trials, limitations such as floor and ceiling effects of some “gold-standard” instruments become an important concern [24–26]. The lack of a common metric across instruments hampers interpretation and comparisons across studies. PROs used in clinical practice to inform medical decision-making may require higher levels of precision and responsiveness than is found in those developed for research purposes in order to reflect changes at the *individual* level. Thus, ideal PROs for use in RA clinical care: a) reflect the aspects of health relevant to people living with the disease [14–16, 27]; b) accurately and precisely measure the symptom or impact across the continuum of disease activity; c) are minimally burdensome to patients and clinicians; d) produce a simple score on a common metric; and e) can be easily interpreted in absolute terms (“*the state*” or severity of the symptom) or as a *change* in terms of improvement and worsening.

The Patient Reported Outcome Measurement Information System (PROMIS®) was developed by the National Institutes of Health (NIH) to provide a standardized metric for measuring physical, mental, and social health across chronic diseases (www.nihpromis.org). PROMIS instruments are publicly available, were developed using item response theory, and have been tested in more than 20,000 individuals drawn from the general US population [28, 29]. Both short forms (SF) and computerized-adaptive tests (CATs) are available to assess common symptoms and function. Results are reported using a common metric (i.e., a T-score with a mean of 50 and standard deviation (SD) of 10) and have been normed to the US population. To date, only the PROMIS *Physical Function* scale has been evaluated in RA [24, 25].

In earlier work, we identified domains that people with RA considered impactful on their health-related quality of life (HRQL) [15, 18]. Here, we describe the performance and validation of 11 PROMIS instruments in adults with RA in the context of ongoing care. We hypothesized that as compared with the general US population, PROMIS scores in people with RA would reflect greater HRQL impairments related to pain, fatigue, sleep, mood, physical function, and participation. We also hypothesized that scores would correlate moderately to strongly with existing legacy instruments assessing similar constructs and would show evidence of a dose-response relationship with disease activity levels.

## Materials and Methods

Data are from a prospective cohort study of people receiving guideline-based RA care in an academic rheumatology clinic. All procedures performed were in accordance with the ethical standards of the institutional and/or national research committee and with the 1964 Helsinki declaration and its later amendments or comparable ethical standards. The study was approved by the Johns Hopkins Institutional Review Board (NA00071923). After providing written informed consent, coordinators registered participants in 94 Assessment Center (www.assessmentcenter.net), a secure online PROMIS research management tool.

### Sample

Adults ages 18+ who were fluent in English and were enrolled in our clinical practice registries were eligible to participate. Exclusions were significant medical or psychiatric illness that the treating clinician felt would limit an individual’s ability to participate in the study.

### Procedures

Individuals were consecutively approached by phone or at the time of a routine clinic visit and provided with details about the study. After providing written informed consent, coordinators registered participants in Assessment Center (www.assessmentcenter.net), a secure online PROMIS research management tool. Assessment Center provides access to the CATs, SFs and study-specific questionnaires, and will automatically generate a report containing scores for PROMIS CATs. After checking in with the clinic receptionist, participants were given a tablet computer linked to a study-specific URL to complete questionnaires described below. RA legacy instruments were also completed. A subset of participants were consecutively approached and asked to complete the same PROMIS measures 2 days later to assess test-retest reliability.

### Measures

#### Sociodemographic and RA Characteristics

Socio-demographic information was drawn from the patient’s medical record and included age, sex, race/ethnicity, education, work status, RA duration, and RF/CCP status. Swollen and tender joint counts (28 joints) and MD global assessments of disease activity (100 mm VAS) were provided by treating rheumatologists. Clinical disease activity index scores (CDAI) were calculated to assess disease activity level.

#### Patient Reported Outcomes

Legacy RA PROs that are routinely collected included the Patient Global Assessment (100 mm VAS), Pain (100 mm VAS), the 8-item Modified Health Assessment Questionnaire of disability (M-HAQ, 0–3 scale), [10] and a fatigue 100 mm VAS [30]; with each measure, higher scores reflect more of the symptom. PROMIS instruments for physical, emotional, and social domains were selected based on earlier work [14–17]. Version 1.0 CATs were administered for: *Pain Interference*, *Fatigue*, *Sleep Disturbance*, *Sleep-Related Impairment*, *Depression*, *Anxiety*, *Anger*, and *Physical Function;* the 3-item PROMIS *Pain intensity* SF was also included. Version 2.0 CATs were administered for *Ability to Participate in Social Roles* and *Satisfaction with Social Roles and Activities*. Specific items, response options, and anchors are available through www.assessmentcenter.net. Scales were administered in fixed order, and CATs were programmed to administer from 4–8 items until a standard error (precision of estimate) fell at or below 0.3. Higher scores indicate more of the trait being measured, so that for physical function, participation, and satisfaction, a higher score is “better”, whereas for symptoms, higher scores indicate higher levels of the symptom.

### Statistical Analysis

Pearson coefficients and Spearman’s rho were used to examine the degree to which PROMIS scores were consistent with each other and legacy PROs for similar domains. ANOVA was used to compare domain scores for PROMIS and legacy variables by CDAI disease activity levels. Pearson correlation and Cronbach’s alpha were used to assess reproducibility and internal consistency; reliability >.7 was considered acceptable. Analyses were done using IBM SPSS version 22.

## Results

### Patient Characteristics

A total of 177 patients were enrolled and completed the surveys between September 2012 and November 2013. The sample reflected a diverse spectrum of sociodemographic and RA characteristics (Table 1). Participants were mostly female (82%) and white (83%) with mean (SD) age of 56 (13) years. Most (92%; 162/177) met ACR2010 criteria for RA [31]. Nearly a quarter (24%) had a high school education or less, and 22% reported being disabled due to RA. Disease duration ranged from 0–41 years; 29% had RA ≤ 5 years with 13% ≤ 2 years. Nearly half (49%; 86/177) were on a biologic, and most (89% 157/177) were on conventional DMARDS, Most patients were in CDAI remission (32%) or low disease activity (LDA: 38%).

**Table 1:** Participant characteristics (N = 177).

Characteristic	N	Value
Age (Mean SD; years)	177	55.5 ± 13.3
Female sex	177	145 (82%)
Race	177	
White		146 (83%)
Black		22 (12%)
Other		9 (5%)
Education	175	
High School		42 (24%)
College		133 (76%)
Employment Status	176	
Full or Part Time		99 (56%)
Not working		19 (11%)
Disabled		39 (22%)
Disabled-not RA		19 (11%)
RA Disease Duration	177	
Mean SD (years)		11.6 ± 9.5
< 2 years		23 (13%)
CDAI (mean; SD)*	176	7.9 ± 7.8
Remission		56 (32%)
Low disease activity		67 (38%)
Moderate disease activity		39 (22%)
High disease activity		14 (8%)
Tender Joint Count (28)	176	1.4 ± 2.9
Swollen Joint Count (28)	175	2.3 ± 3.4
MD Global Assessment (100 mm VAS)	175	13.9 + 15.3
Morning Stiffness		124 (71%)
Yes		124 (71%)
Mean duration (min)		62 ± 87
MHAQ* (mean; SD)	175	0.3 ± 0.4
Pain (100 mm VAS)	175	31 ± 28
Patient Global Assessment (100 mm VAS)	176	29 ± 27
Fatigue (100 mm VAS)	175	40 ± 31

*Modified Health Assessment Scale

### Patient Reported Outcomes

PROMIS and legacy scale scores are shown in Table 2. Mean patient global and pain scores were approximately 30 on a 100-point scale, and fatigue was 40. All legacy PROs were positively skewed. Floor effects were evident with the pain, fatigue and patient global VASs; 26 (15%) reported no pain, 18 (10%) reported no fatigue, and 31 (18%) scored 0 on the Patient Global (very well). MHAQ scores reflected minimal disability with 81 people (46%) scoring 0.

**Table 2:** PROMIS and legacy scale scores in people with rheumatoid arthritis.

	N	Mean	SD	Median	25%	75%	Range	Min	Max	Test-retest r^†^	Cronbach’s alpha (95% CI)
**Patient Global**	176	29.3	27.1	20.0	5.0	50.0	100.0	0	100	--	---
**Physical Function**											
PROMIS Physical Function	177	43.3	9.0	43.0	37.2	48.7	46.0	24.1	70.1	.975	.985 (.981, .988)
**MHAQ** *	175	0.3	0.4	0.1	0.0	0.5	1.8	0.00	1.75	--	--
**Pain**											
PROMIS Pain Intensity	177	44.6	8.2	45.7	40.5	51.3	31.4	30.7	62.1	.778	.906 (.879, .928)
PROMIS Pain Interference	177	53.7	9.5	55.8	46.6	60.6	34.6	39.1	73.7	.880	.990 (.988, .992)
*Pain 100mm VAS*	175	*39*.*6*	*28*.*3*	*25*.*0*	*5*.*0*	*50*.*0*	*100*.*0*	*0*	*100*	*--*	*--*
**Fatigue**											
PROMIS Fatigue	177	53.9	10.0	53.5	48.5	62.1	49.7	26.3	76.0	.883	.988 (.985, .991)
**Fatigue 100mm VAS**	175	39.7	30.9	35.0	10.0	70.0	100.0	0	100	--	--
**Additional PROMIS Scales**											
Depression	176	49.1	8.8	49.9	43.7	54.3	36.7	34.9	71.6	.859	.983 (.978, .987)
Anxiety	176	50.9	8.1	51.2	46.2	56.0	37.5	33.6	71.1	.822	.970 (.961, .976)
Anger	176	47.3	9.1	46.8	41.9	52.8	51.8	29.4	81.2	.822	.978 (.972, .983)
Sleep Disturbance	177	51.5	9.8	52.0	44.7	57.9	46.3	27.5	73.8	.831	.987 (.983, .990)
Sleep Impairment	177	51.6	9.5	52.5	44.8	57.4	52.2	26.8	79.0	.725	.978 (.972, .983)
Ability to Participate Social	175	50.1	9.0	51.2	43.8	56.3	44.0	22.4	66.4	.747	.985 (.981, .989)
Satisfaction Social Participation	175	49.0	10.2	49.2	41.3	57.4	42.7	24.5	67.2	.775	.990 (.988, .992)

*Modified Health Assessment Questionnaire. All PROMIS measures are computerized adapted tests, except the Pain Intensity 3a which is a short form.

^†^All p’s < .001. Mean (SD) 2.2 (0.6) days apart. N = 34.

**Fig 1:**
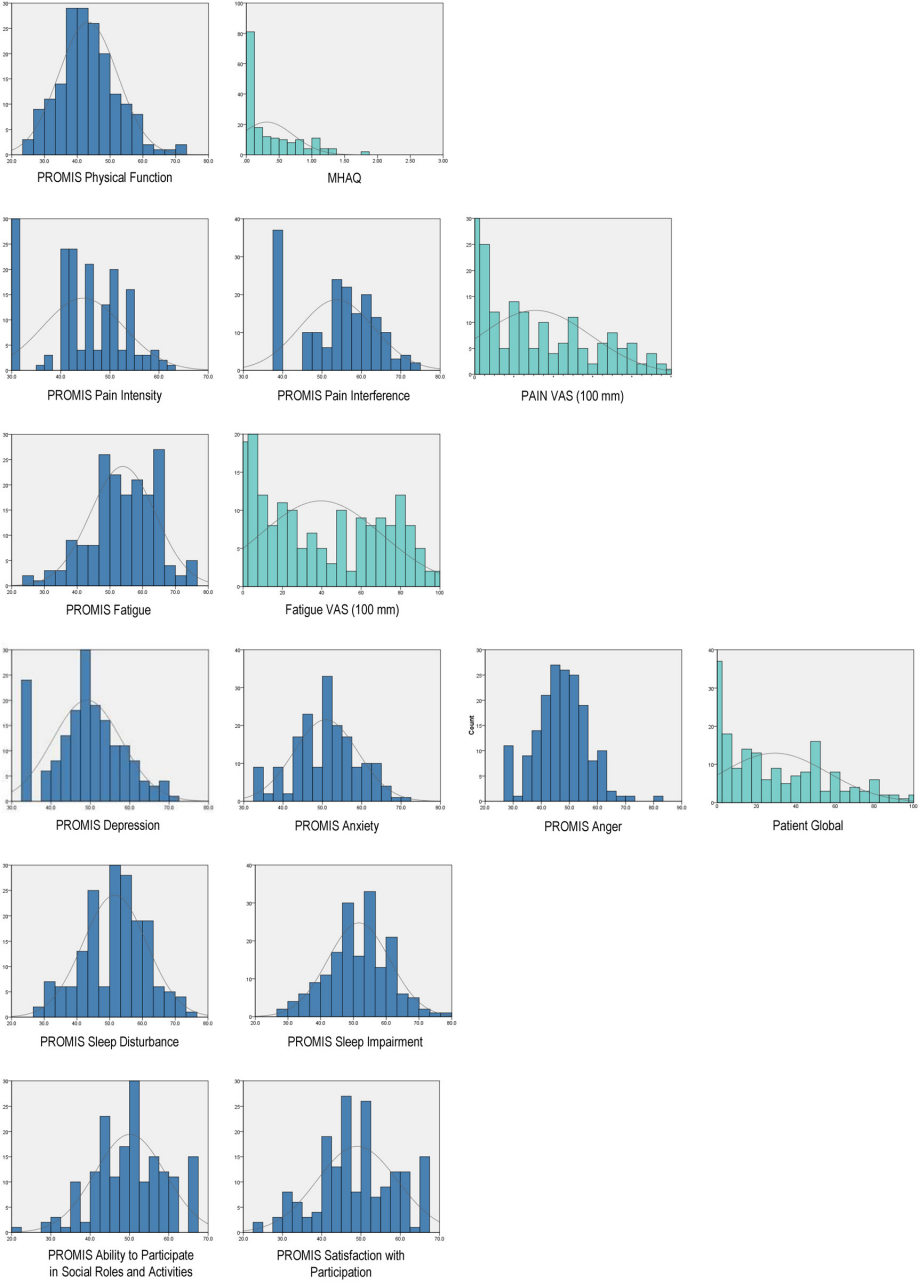
Distribution of PROMIS and legacy scores in rheumatoid arthritis sample (N = 177).

PROMIS scores were distributed across a broad range (i.e., -2.7 to +3.1 SD; see Fig 1) for each PROMIS measure, and were relatively normally distributed except *Pain Intensity*, *Pain Interference* and *Depression* which showed positive skewing. Across PROMIS instruments, mean scores were between 45 and 55 (i.e., within normal limits or 0.5 SD of US general population norms) except for *Physical Function* and *Pain Intensity* which were significantly lower than population norms. Across CATs, the median number of items administered was 3, except for *Anger* which was 4, and median completion time was 7 minutes.

#### Reliability

Among the 34 participants who completed PROMIS measures 2.2 (0.6) days later, correlations ranged from .725 *(Sleep-related Impairment)* to .975 *(Physical Function)*, with 7 scales ≥ .822 (Table 2). Cronbach’s alpha showed high internal consistency ranging from .906 (*Pain Intensity 3a*) to .988 (*Fatigue*).

#### Correlations among PROMIS Measures

Correlations among individual PROMIS scales ranged from weak to strong (e.g., r’s 0.23 to 0.85; all p’s ≤.002) (Table 3). The highest correlations (≥ 0.7) were evident among scales measuring similar constructs: physical health (e.g., pain, fatigue, sleep), mental health (e.g., depression, anxiety, anger), and social health (*Ability to Participate in Social Roles* and *Satisfaction with Social Roles and Activities*). *Physical Function* was also strongly correlated with *Pain Interference* (r = -0.71) and *Ability to Participate in Social Roles* (r = 0.70). The two participation scales were moderately to strongly (r’s-.34 to .70) correlated with all symptom and function scales.

**Table 3:** Correlations among PROMIS scales.

	Physical Function	Pain Intensity	Pain Interfere	Fatigue	Sleep Disturb	Sleep Impair	Depress	Anxiety	Anger	Ability to Participate Social	Satisfaction with Role Activities
Physical Function	---	-.561	-.709	-.635	-.376	-.432	-.398	-.361	-.229	.698	.627
Pain Intensity	-.561	---	.854	.647	.493	.533	.421	.437	.324	-.509	-.544
Pain Interference	-.709	.854	---	.672	.480	.547	.521	.515	.394	-.640	-.618
Fatigue	-.635	.647	.672	---	.453	.670	.487	.523	.407	-.607	-.581
Sleep Disturbance	-.376	.493	.480	.453	---	.719	.394	.401	.296	-.341	-.389
Sleep Impairment	-.432	.533	.547	.670	.719	---	.573	.616	.507	-.461	-.434
Depression	-.398	.421	.521	.487	.394	.573	---	.802	.761	-.541	-.517
Anxiety	-.361	.437	.515	.523	.401	.616	.802	---	.737	-.524	-.463
Anger	-.229	.324	.394	.407	.296	.507	.761	.737	---	-.431	-.341
Ability to Participate	.698	-.509	-.640	-.607	-.341	-.461	-.541	-.524	-.431	---	.744
Satisfaction Roles Activities	.627	-.544	-.618	-.581	-.389	-.434	-.517	-.463	-.341	.744	---
CDAI	-.593	.537	.540	.537	.312	.352	.311	.280	.220	-.495	-.502

All Pearson correlation coefficients were statistically significant (p≤.01). All PROMIS measures are computerized adapted tests, except the Pain Intensity 3a which is a short form.

#### Convergent and Known Groups Validation with Legacy Instruments

The PROMIS *Physical Function*, *Pain Intensity* and *Pain Interference* and *Fatigue* instruments correlated strongly (rho’s ≥ 0.75;p’s ≤ 0.01) with corresponding legacy instruments (Table 4). Patient Global was moderately to strongly (rho’s ≥ 0.68; p’s ≤ 0.01) associated with PROMIS scales. The lowest associations were between Patient Global and PROMIS mood scales (*Anger*, rho = 0.32; *Depression*, rho = 0.41, and *Anxiety*, rho = 0.41; all p’s ≤ 0.01).

**Table 4:** Correlations between PROMIS and legacy measures in RA.

	MHAQ	PROMIS Physical Function	Pain VAS	PROMIS Pain Intensity	PROMIS Pain Interfere	Fatigue VAS	PROMIS Fatigue	Patient Global VAS
MHAQ	1.000	-.752	.514	.541	.655	.493	.506	.593
PROMIS Physical Function	-.752	1.000	-.593	-.601	-.749	-.570	-.645	-.688
Pain VAS	.514	-.593	1.000	.856	.823	.704	.642	.842
PROMIS Pain Intensity	.541	-.601	.856	1.000	.842	.651	.665	.759
PROMIS Pain Interference	.655	-.749	.823	.842	1.000	.654	.690	.770
Fatigue VAS	.493	-.570	.704	.651	.654	1.000	.862	.739
PROMIS Fatigue	.506	-.645	.642	.665	.690	.862	1.000	.682
Patient Global VAS	.593	-.688	.842	.759	.770	.739	.682	

All Pearson correlation coefficients were statistically significant at p ≤ 0.01.

All PROMIS measures are computerized adapted tests, except the Pain Intensity 3a which is a short form.

In general, PROMIS scores worsened significantly (p < .05) as disease activity increased from remission through high disease activity (Table 5); physical health scores worsened by 12–17 points, social domains by 16–18 points, and emotional health by 8–11 points. A dose-response relationship was evident in *Physical Function*. Similar trends were evident in all scales, although scores were not significantly different between low and moderate disease activity levels for most measures, except *Anger*, which remained within normal limits for remission, low, and moderate disease activity and worsened only in those with high disease activity. In all PROMIS physical and social health instruments, increases in impairment were highest between people in LDA vs. remission (≥ 0.7 SD); *Anxiety* and *Depression* worsened by nearly 0.5 SD, while *Anger* increased only slightly. Similar patterns were seen between those in high vs. moderate disease activity, where impairment increased on average 0.5 SD; the exception was *Pain Intensity*, where scores increased an average of 3.4 points.

**Table 5:** PROMIS and legacy scores by CDAI disease activity levels.

	Remission (n = 56)		Low (n = 67)*		Moderate (n = 39)^†^		High (n = 14)	
	Mean	SD	Mean	SD	Mean	SD	Mean	SD
***Physical Function***								
PROMIS Physical Function	50.1_a_	8.8	42.2_b_	7.1	39.2_c_	5.9	32.9_d_	5.5
*MHAQ*	.*1*_*a*_	.*3*	.*3*_*b*_	.*3*	.*5*_*c*_	.*4*	.*8*_*d*_	.*5*
***Pain***								
PROMIS Pain Intensity	37.6_a_	6.6	46.4_b_	6.4	48.8_b,c_	6.4	52.1_c_	7.8
PROMIS Pain Interfere	45.6_a_	7.2	56.0_b_	8.3	57.8_b_	6.1	63.4_c_	8.6
*Patient Pain VAS*	*5*.*8*_*a*_	*7*.*6*	*35*.*5*_*b*_	*26*.*2*	*49*.*9*_*c*_	*25*.*3*	*55*.*3*_*c*_	*25*.*6*
***Fatigue***								
PROMIS Fatigue CAT	46.2_a_	8.6	55.7 _b_	8.3	58.5_b_	6.9	64.0_c_	9.6
*Fatigue VAS*	*14*.*1*_*a*_	*19*.*2*	*46*.*3*_*b*_	*28*.*7*	*55*.*0*_*b*,*c*_	*28*.*0*	*66*.*6*_*c*_	*20*.*6*
***Additional Domains***								
PROMIS Depression	45.7_a_	7.9	50.1_b_	8.5	50.1_b_	8.8	56.2_c_	8.2
PROMIS Anxiety	47.6_a_	7.3	52.2 _b_	8.5	51.3_b_	7.0	57.0_c_	7.8
PROMIS Anger	45.3_a_	7.9	47.4_a_	9.6	48.0_a,b_	9.4	52.9_b_	8.8
PROMIS Sleep Disturbance	46.6_a_	8.6	53.4_b,c_	8.9	52.9_b_	10.7	58.7_c_	7.9
PROMIS Sleep Impairment	46.4_a_	8.6	53.7_b_	9.6	52.9_b_	8.1	59.4_c_	6.6
PROMIS Ability to Participate Social	55.8_a_	8.3	49.1_b_	7.8	47.8_b_	6.8	38.8_c_	6.8
PROMIS Satisfaction with Role Activities	55.8_a_	8.7	47.7_b_	9.8	45.4_b_	6.8	37.1_c_	7.3
*Patient Global VAS*	*5*.*3*_*a*_	*6*.*7*	*29*.*3*_*b*_	*22*.*7*	*52*.*0*_*c*_	*21*.*0*	*61*.*1*_*c*_	*25*.*2*

Note: Values in the same row not sharing the same subscript are significantly different at p< .05. SD = Standard deviation. All PROMIS measures are computerized adapted tests, except the Pain Intensity 3a which is a short form.

*N = 66 for MHAQ, Pain VAS and Fatigue VAS;

^**†**^N = 38 for Depression, Anxiety, Anger, and Ability to Participate Social scales.

## Discussion

This study is the first to report evidence of the reliability and construct validity of 11 PROMIS instruments in people with RA within the context of ongoing care. We selected PROMIS instruments that reflect outcomes people with RA identified as important to them in our foundational work that included a literature review, focus groups with patients, surveys of experts, and combined patient-provider consensus Delphi exercises [15, 16, 32]}. The 11 instruments were completed in <11 minutes by 75% of patients. *Pain (Intensity* and *Interference)*, *Physical Function* and *Fatigue* scores correlated highly (rho’s ≥ 0.75) with corresponding legacy measures. A dose-response relationship was evident across disease activity levels for the *Physical Function*, *Pain* and *Fatigue* scales. Additional domains we examined including mood, sleep, and participation also showed similar trends. These findings contribute new evidence supporting the feasibility and construct validity of PROMIS when comparing RA with other diseases, as outcomes in comparative effectiveness trials, and for RA clinical care.

The process for developing and validating PROs has evolved considerably over the last two decades and now includes recommendations to identify patient-relevant symptoms through qualitative inquiry, cognitively test and debrief of potential items, rigorously psychometrically evaluate, and validate in the targeted patient population and context of intended use (e.g. RCT vs. clinical practice) [3, 23, 33]. PROMIS instruments were initially developed to help researchers obtain precise estimates of symptoms and functional impacts from patients across chronic diseases using a common metric. Although PROMIS was developed and tested in the general US population and later in selected clinical conditions, evaluating the content validity of instruments, construct validity against legacy instruments, and the responsiveness of these instruments in specific conditions is necessary as outlined in the PROMIS instrument maturity model [23].

An important strength of the PROMIS instruments was the ability to capture the experiences using a common T-score metric and across the broad continuum of symptoms and function experienced by people with RA spanning roughly ± 3 SD (or 99.7% of data in a normal distribution). Notably, fatigue, emotional distress, sleep, and participation, which are not currently part of the recommended RA core set [30], also showed a wide distribution of scores, with many individuals reporting significant impairments. Floor and ceiling effects, recognized limitations of many instruments [24, 25], were evident for many patients with legacy measures; for example, in our sample nearly 1 in 2 (46%) scored 0 on the MHAQ. Among the 56 people in remission, substantial proportions of individuals scored 0 on legacy instruments of pain (41%), physical function (75%), fatigue (27%) and patient global (46%). In contrast, PROMIS scores for people in remission showed considerable dispersion; the range for *Pain intensity* was 22 points (T-scores of 31 to 52), 43 points for *Physical Function* (27 to 70) and *Satisfaction with Role Activities* (24 to 67), and 44 points (22 to 66) for *Ability to Participate in Social Roles and Activities*. In physical and social domains, the largest increase in impairment was between people in remission and those in LDA. Conversely, in emotional domains, minimal differences were seen among lower levels of disease activity (remission to low, low to moderate), with the greatest differences evident between moderate and high disease activity. Most scores on PROMIS measures were higher in people with moderate vs. low disease activity, though differences were not statistically significant. However, the relatively small number of patients in each group and significantly clustering of individuals around the cut point between low and moderate disease activity may have contributed to this finding.

Reliable, precise, and accurate measurement of symptoms and functional impacts across the continuum of disease activity has never been more important to optimize RA treatment given that remission or LDA is the current target for management.[11, 34]. With the development of biologics and the focus on early, intensive treatment, many people with RA now reach states of remission or LDA; in our sample, 69% were at these targets. Composite RA disease activity measures, (e.g. Disease Activity Score [DAS28], CDAI, Simplified Disease Activity Index [SDAI]) rely on the answer to a single global question about disease activity or health status. However, multidimensional measures such as the SF-36 are proprietary and burdensome to complete and score in clinical practice settings. From the battery of instruments available, we were able to select PROMIS instruments to focus on important outcomes that either can only come from patients (i.e., symptoms) or those that are most practical to obtain by asking patients (impacts). PROMIS CATs offer optimal precision on a common metric with immediate scoring for real time use in clinical encounters. The ability of PROMIS to detect small changes even at the low end of symptoms and disability in patients with minimal disease activity can offer new insight into the relative burden of living with RA and new opportunities to compare the impact and side effects associated with current treatments, as well as the ability to capture changes in HRQL that may be relevant to tapering therapies after achieving a target of remission.

Findings from domains in which we assessed both symptom intensity and impact (e.g., *Pain*, *Sleep*, and *Participation*) produced some discrepancies that were not expected. Median *Pain Interference* scores were 10 points higher than *Pain Intensity*, suggesting the impact of pain on day-to-day function may be much greater than what scores on *Pain Intensity* scores reflect. Among patients in remission (CDAI <2.8), 36 (64%) had CDAI scores ≤ 1.0, supporting the absence of detectable disease and “deep” remission. Within this group, median T-scores were better than population norms for *Pain Intensity* (30.7), *Pain Interference* (39.1), *Sleep Impairment* (44.5), *Depression* (43.6), *Anger* (44.1), *Ability to Participate Social* (59.1) *and Satisfaction with Social Roles* and *Participation* (58.3); *Physical Function* was at the population norm (50.7). Higher scores may indicate a response shift reflecting how patients adapt to and report their level of symptoms and function over time [35]. Response shifts occur as patients reconceptualize their life circumstances, reprioritize what is important, and recalibrate (e.g., what pain scores of 10 represent) as they learn to live with RA [36]. For instance, some RA patients have reported that when they record a score of “0” on a questionnaire, this does not necessarily represent the absence of a symptom, but instead reflects a new baseline of “what is normal for me” [37]. Thus, our findings also raise important questions in defining the expected “norms” for RA symptoms and function. Further evaluation in larger numbers of patients across the continuums of age, disease activity, duration, disability, and adaptation is warranted to define RA norms.

Before widespread use of PROMIS in RA research and care can be recommended, it will be important to evaluate their performance in relevant subgroups and demonstrate that the instruments are sufficiently responsive or sensitive to change over time. Evaluation of how PROMIS scores change with fluctuations in disease activity is needed to define minimally detectable differences and clinically meaningful changes, essential parameters to facilitating their use in longitudinal care for individuals. Whether the PROMIS instruments perform similarly in other forms of arthritis, autoimmune, and inflammatory diseases remains to be determined.

Strengths of this study include use of a well-characterized cohort with the broad range of characteristics reflective of patients seen in real world settings. We evaluated the performance of PROMIS instruments within the context of usual care. Limitations of the study include use of a mostly white, well-educated sample with established RA that was generally well controlled. In our study, participants were English-speaking; there are ongoing efforts to evaluate translated versions of PROMIS instruments and examine cross-cultural validity [38]. The legacy measures used in this study were limited to ACR core set PROs that we routinely administer; PROMIS anxiety, depression and anger measures already have strong evidence of validity with cross-walks available for legacy measures [39–41]. We used PROMIS CATs which require an internet connection to Assessment Center and may not be feasible in some settings. The use of SFs in RA clinical care warrants further study to determine whether these retain sufficient precision for clinical decision-making [42].

## Conclusions

This study contributes new evidence supporting the reliability and construct validity of 11 PROMIS instruments in RA and feasibility of real-time administration and scoring for use in clinical practice. Results demonstrate the considerable impact that RA may have on multiple domains of physical, emotional, and social health. This work provides important preliminary data supporting the applicability of PROMIS in RA research and care with broad implications for other forms of inflammatory and autoimmune diseases in estimating the intensity and impact of symptoms and function important to patients. Ongoing validation of the ‘universal’ PROMIS instruments in specific diseases such as RA can facilitate comparisons across diseases, treatments, cultures, and countries.
